# Supplementary material for: Genomic 8-oxoguanine modulates gene transcription independent of its repair by DNA glycosylases OGG1 and MUTYH
Source: Redox Biol. 2024 Dec 5;79:103461. doi: 10.1016/j.redox.2024.103461 (PMC11697278; doi:10.1016/j.redox.2024.103461)
Supplement: Multimedia component 6 [file mmc6.docx]

SUPPLEMENTARY DATA

**Genomic 8-oxoguanine modulates gene transcription independent of its repair by DNA glycosylases OGG1 and MUTYH**

Tobias Obermann, Teri Sakshaug, Vishnu Vignesh Kanagaraj, Andreas Abentung, Mirta Mittelstedt Leal de Sousa, Lars Hagen, Antonio Sarno, Magnar Bjørås, and Katja Scheffler

Supplementary Table 1 - 3

Supplementary Figure 1 - 7

**Supplementary Table 1: CRISPR/Cas9-induced InDels of *OGG1* and *MUTYH*.**
The length of the insertion (In) or deletion (Del) is indicated.

| **Target gene** | **Product number** | **Clone** | **Parental clone** | **Target exon** | **Guide RNA** | **InDel** |
| --- | --- | --- | --- | --- | --- | --- |
| *OGG1* | HZGHC000424c015 | 1 | WT HAP1 | 1 | GTACGATGCCCCATGCGCCT | 5 bp Del |
|  | HZGHC000424c022 | 2 | WT HAP1 | 1 | GTACGATGCCCCATGCGCCT | 8 bp Del |
| *MUTYH* | HZGHC000788c007 | 1 | WT HAP1 | 3 | CTTGGTCGTACCAGCTTAGC | 13 bp Del |
|  | HZGHC000788c008 | 2 | WT HAP1 | 3 | CTTGGTCGTACCAGCTTAGC | 5 bp Del |
| *OGG1* and *MUTYH* (DKO) | NA | 1 | OGG1 clone 2 | 1 | GTACGATGCCCCATGCGCCT | 8 bp Del |
|  |  |  |  | 3 | CTTGGTCGTACCAGCTTAGC | 1 bp Ins |
|  | NA | 2 | OGG1 clone 2 | 1 | GTACGATGCCCCATGCGCCT | 8 bp Del |
|  |  |  |  | 1 | CTTGGTCGTACCAGCTTAGC | 5 bp Del |

**Supplementary Table 2: Reported and observed global GC content of OG enriched regions from OG-seq**

|  | **Average GC-content** |
| --- | --- |
| Reported global | 40.9 % |
| WT | 49.9 % |
| *MUTYH^-/^* | 53.5 % |
| *OGG1^-/^* | 56.3 % |
| DKO | 50.1 % |

**Supplementary Table 3: GC content of different genomic features used in regional analysis**

| **Region** | **Average GC-content** |
| --- | --- |
| Intergenic | 46.8% |
| Upstream | 48.3% |
| Introns | 45.9% |
| Downstream | 43.8% |
| CpG island | 68.7% |
| Exons | 50.8% |
| G4 | 63.0% |


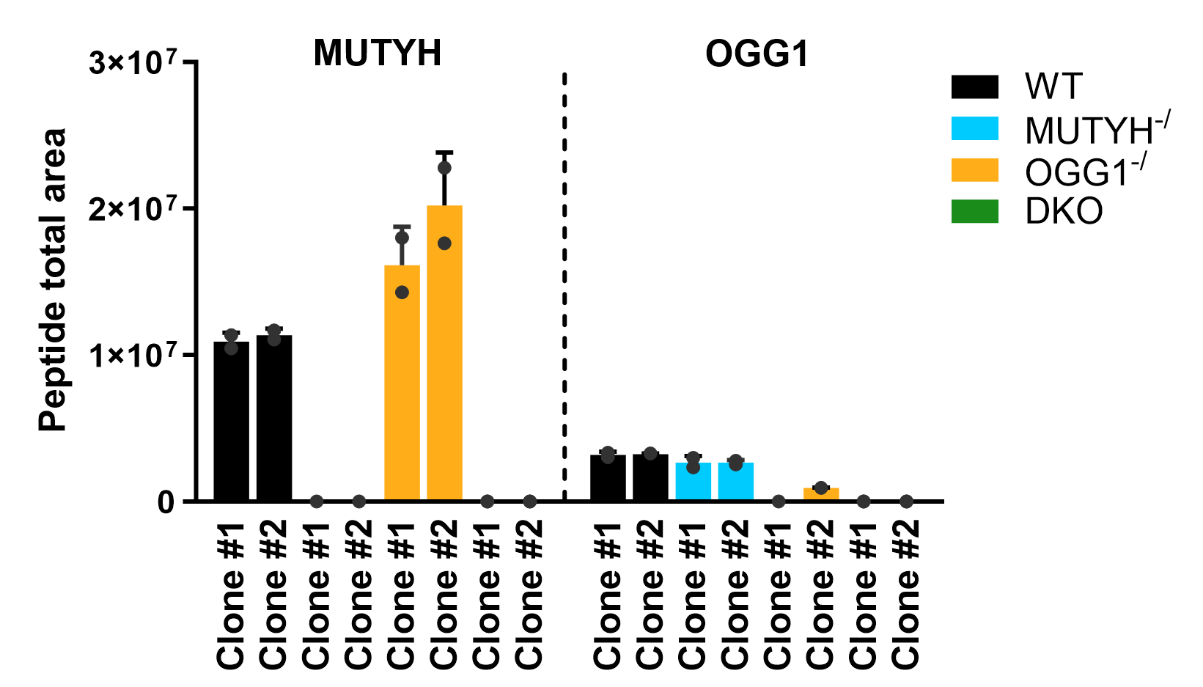


Supplementary Figure 1: Levels of DNA glycosylases MUTYH and OGG1 in HAP1 cells separated by clones measured by targeted mass spectrometry.


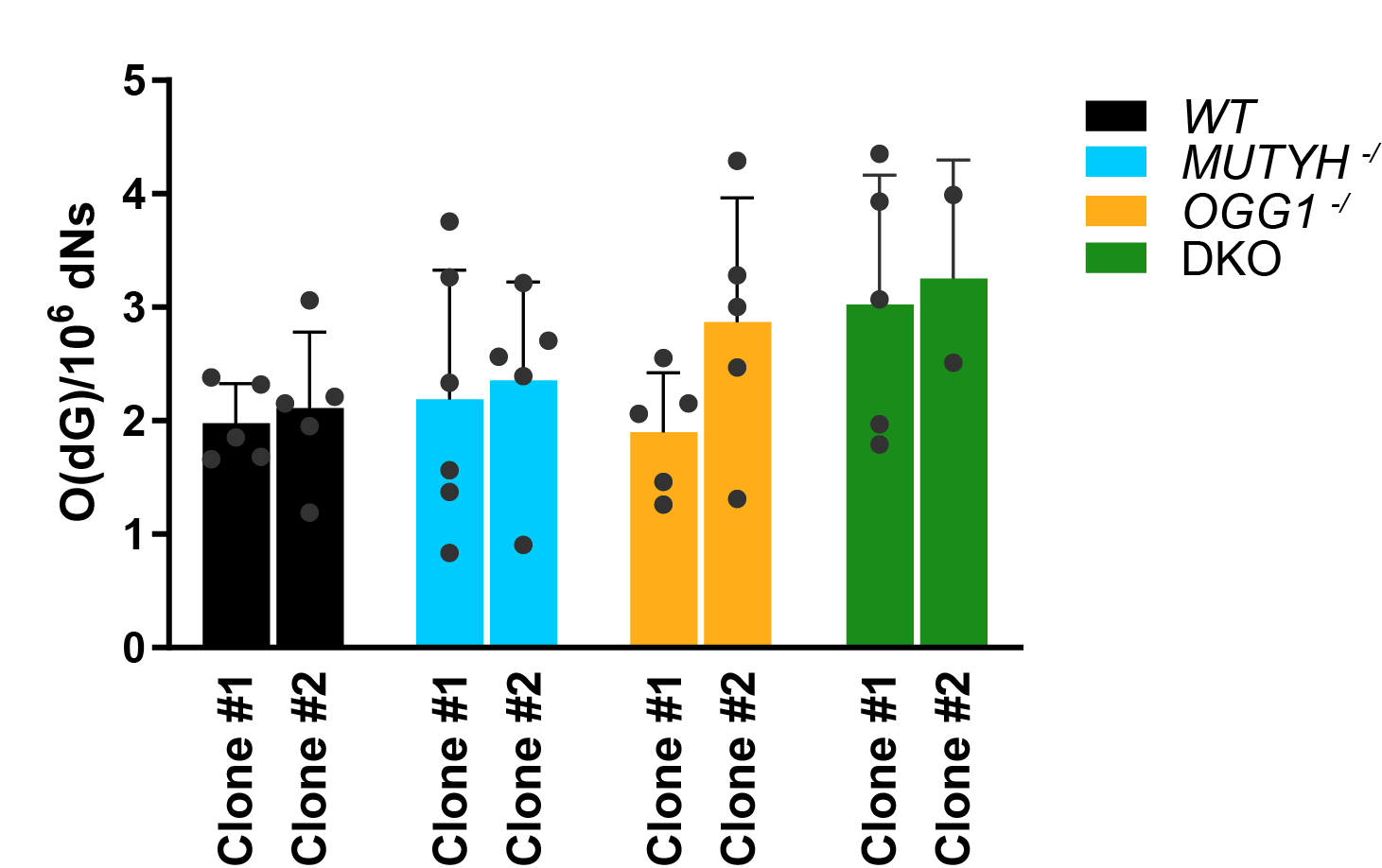


Supplementary Figure 2: Global levels of single nucleotide 8-oxoguanine (O(dG)) per deoxyribonucleosides (dNs) of naïve cells, separated by clones. No significant differences were found. Statistical analyses were done in GraphPad Prism software 10.1 using 2-way ANOVA with Dunnett’s multiple comparisons test. P-values < 0.05 were considered significant. Error bars represent standard deviation from the mean.


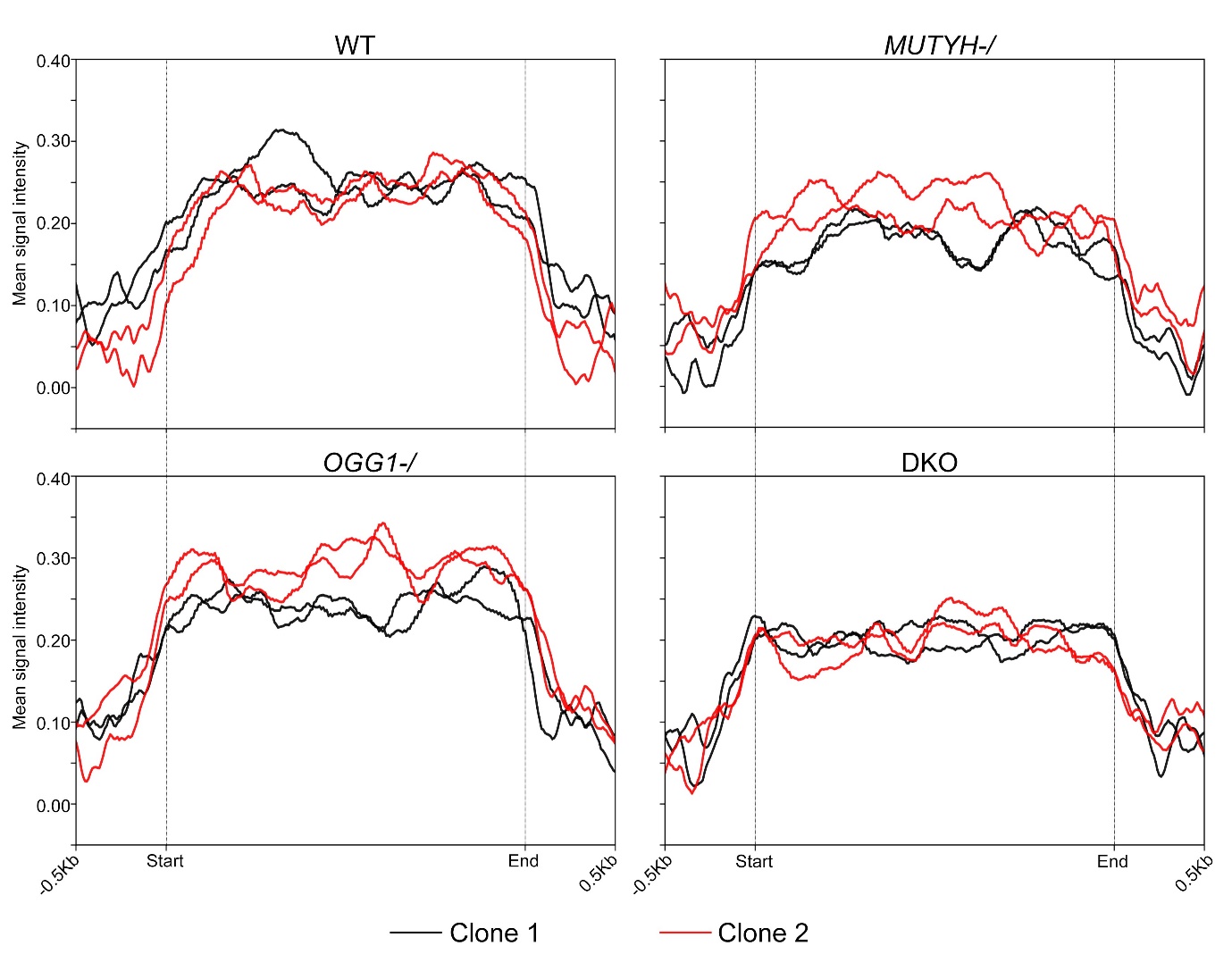


Supplementary Figure 3: Profile plots of average signal intensity of clones show no clonal differences in OG-coverage.


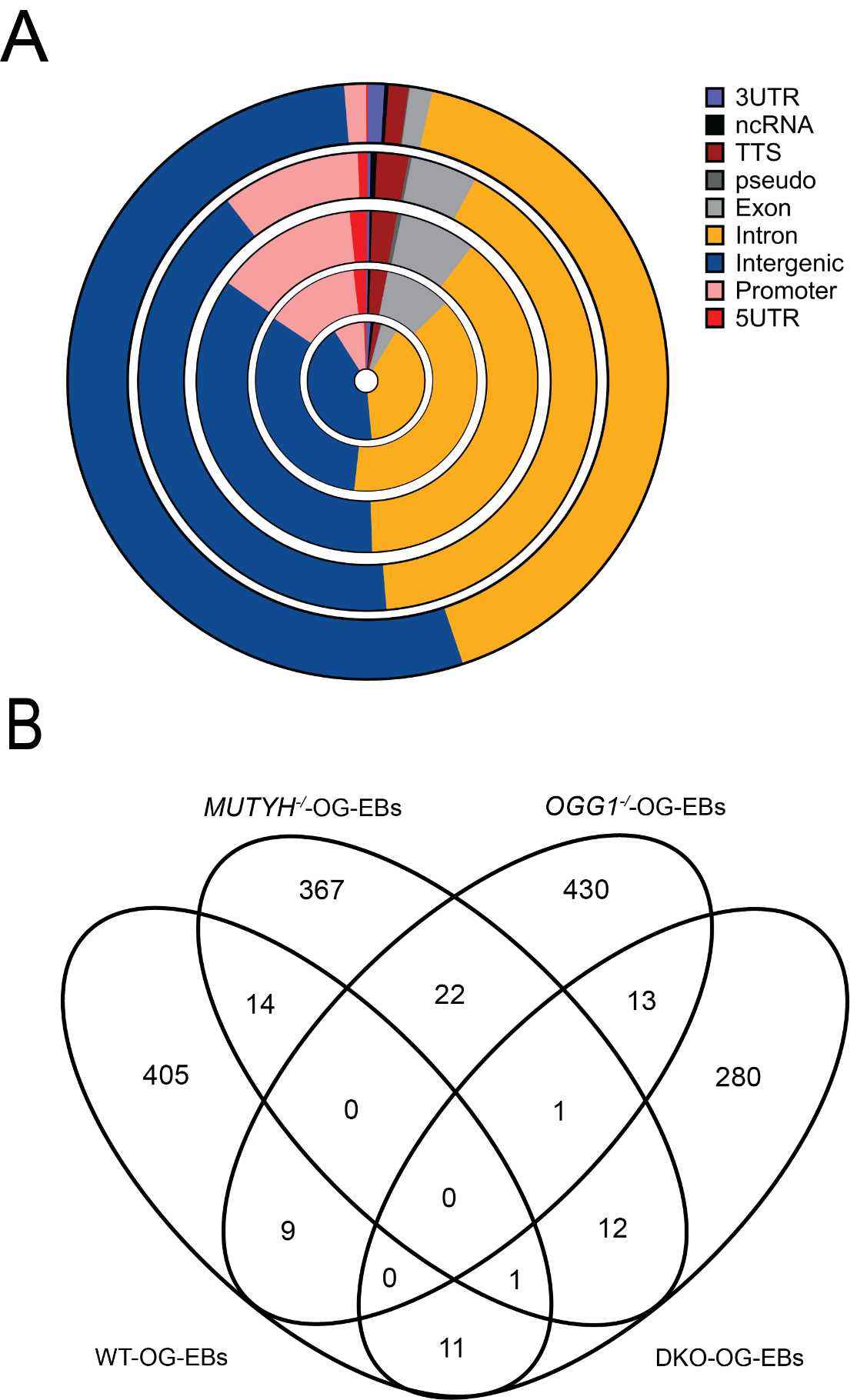


Supplementary Figure 4: Genomic distribution of OG enriched bins and overlap between genotypes. (A) Distribution of OG-enriched bins (OG-EBs) in different genomic features. Outer Ring towards inner ring: expected over the whole genome, WT, *MUTYH^-/^*, *OGG1*^-/^ and DKO. (B) Overlap of annotated genes in OG-EBs across the genotypes.


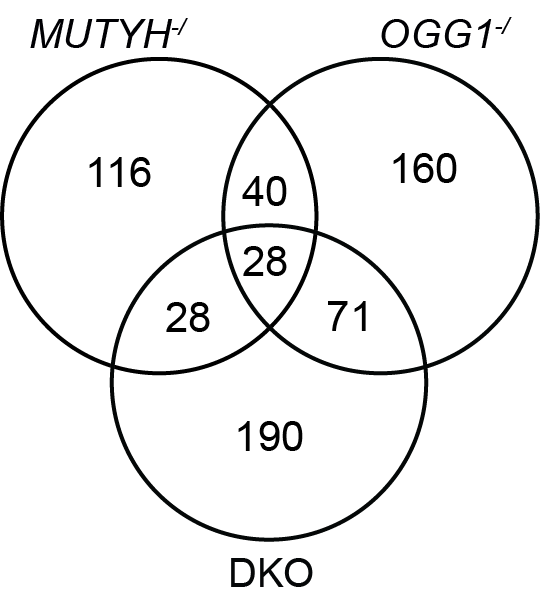


Supplementary Figure 5: Overlap of differentially expressed genes in *MUTYH^-/^*, *OGG1*^-/^ and DKO as identified by RNA-seq.


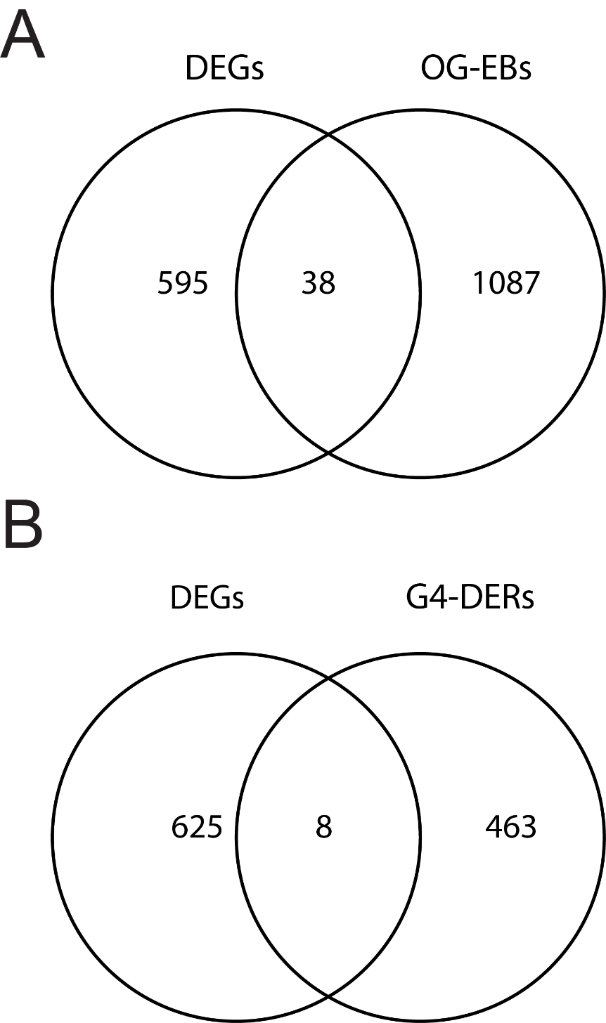


Supplementary Figure 6: Overlap of differentially expressed genes (DEGs) across all genotypes with (A) OG enriched bins (OG-EBs) and (B) OG-differentially enriched regions within G4 motif regions (G4-DERs) identified in the KOs.


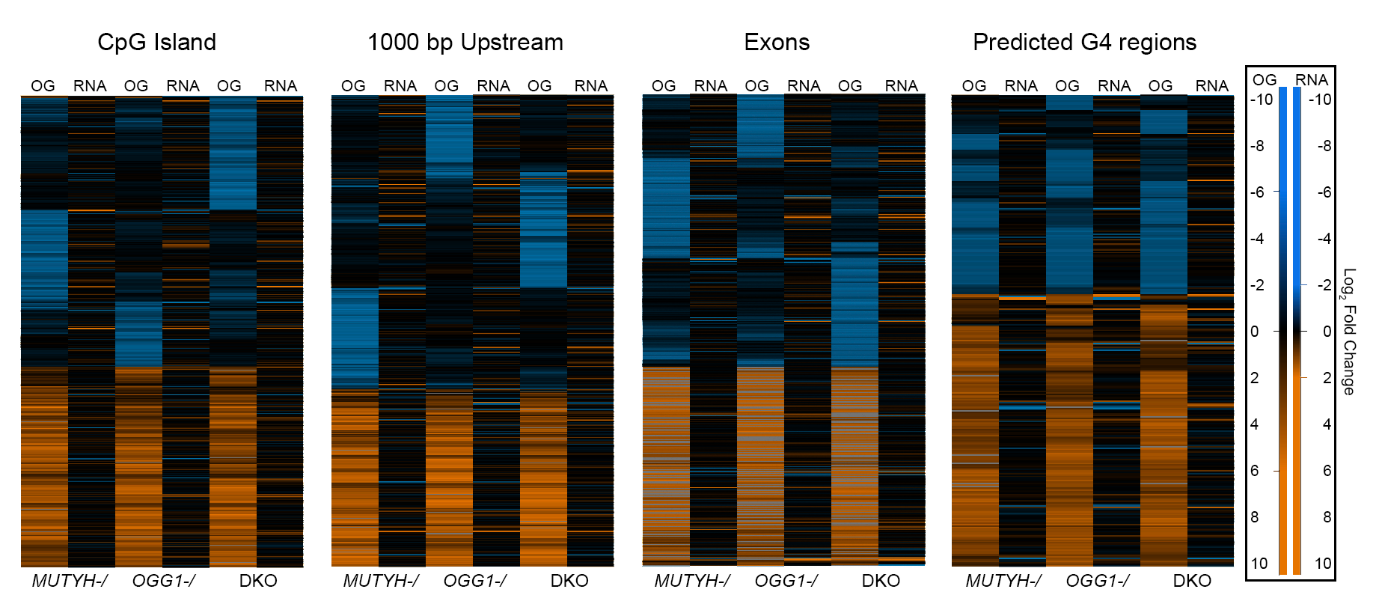


Supplementary Figure 7: Comparison of RNA-seq and OG-seq in different genomic features across DNA glycosylase deficient cells. Clustered heatmaps showing log_2_ fold change of differentially enriched regions (OG-DERs) in OG sequencing and corresponding log_2_ fold change of RNA sequencing across *MUTYH^-/^*, *OGG1^-/^* and DKO HAP1 cells. Blue indicates lower OG accumulation and lower gene expression compared to the WT control, while orange indicates increased OG accumulation or gene expression.
